# Development and internal validation of a prediction model for the presence of tinnitus in a Dutch population-based cohort

**DOI:** 10.3389/fneur.2023.1213687

**Published:** 2023-08-03

**Authors:** Maaike M. Rademaker, Adriana L. Smit, Robert J. Stokroos, Maarten van Smeden, Inge Stegeman

**Affiliations:** ^1^Department of Otorhinolaryngology and Head and Neck Surgery, University Medical Center Utrecht, Utrecht, Netherlands; ^2^UMC Utrecht Brain Center, Utrecht University, Utrecht, Netherlands; ^3^Julius Center for Health Sciences and Primary Care, University Medical Centre Utrecht, Utrecht University, Utrecht, Netherlands

**Keywords:** tinnitus, prediction, prediction model, hearing, epidemiology

## Abstract

**Objectives:**

In this study we aim to develop and internally validate a prediction model on tinnitus experience in a representative sample of the Dutch general population.

**Methods:**

We developed a multivariable prediction model using elastic net logistic regression with data from the Dutch Lifelines Cohort Study. This is a multigenerational cohort study on adults who are located in the northern parts of the Netherlands. The model was internally validated using 10-fold cross-validation. The outcome of the model was tinnitus presence, for which we used 24 candidate predictors on different domains (among others demographic, hearing specific, and mental health variables). We assessed the overall predictive performance, discrimination, and calibration of the model.

**Results:**

Data on 122.884 different participants were included, of which 7,965 (6.5%, 0 missing) experienced tinnitus. Nine variables were included in the final model: sex, hearing aids, hearing limitations, arterial blood pressure, quality of sleep, general health, symptom checklist of somatic complaints, cardiovascular risk factors, and age. In the final model, the Brier score was 0.056 and 0.787 in internal validation.

**Conclusion:**

We developed and internally validated a prediction model on tinnitus presence in a multigenerational cohort of the Dutch general population. From the 24 candidate predictors, the final model included nine predictors.

## Introduction

Tinnitus is a heterogeneous condition that manifests itself differently, in terms of the etiology of the disease, different courses, and comorbidities (1, 2). The concept of tinnitus consists of two components: the sole sensory component, which can be expressed in terms of loudness, frequency, or pitch, and an affective component, which reflects the patient’s emotional reaction and related suffering. The first is referred to as tinnitus and the second as tinnitus disorder (3). Considering the prevalence of tinnitus, a recent meta-analysis showed that the pooled prevalence of any type of tinnitus in adults was 14.4% (95% CI 12.6–16.5%), which results in approximately 740 million people globally (4). The high prevalence and chronicity of tinnitus lead to ample socioeconomic costs. For example, in the Netherlands, the average societal costs are approximated at €6.8 billion per year (5). Healthcare costs for tinnitus alone are estimated at 1.9 billion euros in the Netherlands, at £750 million per year in Great Britain, and at $660 per patient annually in the United States (5–7). Identifying and predicting which people are at a higher risk of developing tinnitus could help to design preventive measures and dedicate healthcare programs for those at risk. These might improve quality of life and reduce costs.

The literature on associations with tinnitus experience is elaborate. Associations between experiencing tinnitus and otologic risk factors but also among others, demographic, cardiovascular, dietary, psychological, and neurological risk factors have been studied (8). However, in a recent systematic review, hearing loss, occupational noise exposure, otitis media, diabetes, temporomandibular disorder, and ototoxic platinum exposure were identified as the most reliable associations (9). Additionally, prediction models can provide individual risk estimates and can inform decision-making in the clinical setting (10). In a recent systematic review of our research group, we identified four prediction models for assessing tinnitus presence. While the sample sizes of these studies were sufficient (*n* = 4,950 to 168.348 per study), the statistical analyzes were often a source of bias (11).

To produce a reliable prediction model that is useful in clinical settings the development of a prediction model should be based on three phases (10). The first phase is the model derivation phase. This includes the identification of predictors and fitting of the model. In the second phase, the model validation phase, the performance of the model is evaluated. In this stage internal validation is used to evaluate the performance of the prediction model. Lastly, one should assess the impact of the model (10). It is essential to adhere to this methodology and properly report these steps in order to produce high-level, clinically useful models.

Based on the high prevalence of tinnitus in the general population, its impact, and related societal and healthcare costs, we aimed to develop and internally validate a prediction model on tinnitus experience in a representative sample of the Dutch general population.

## Methods

This study was reported in accordance with the TRIPOD statement (12).

**Figure 1:**
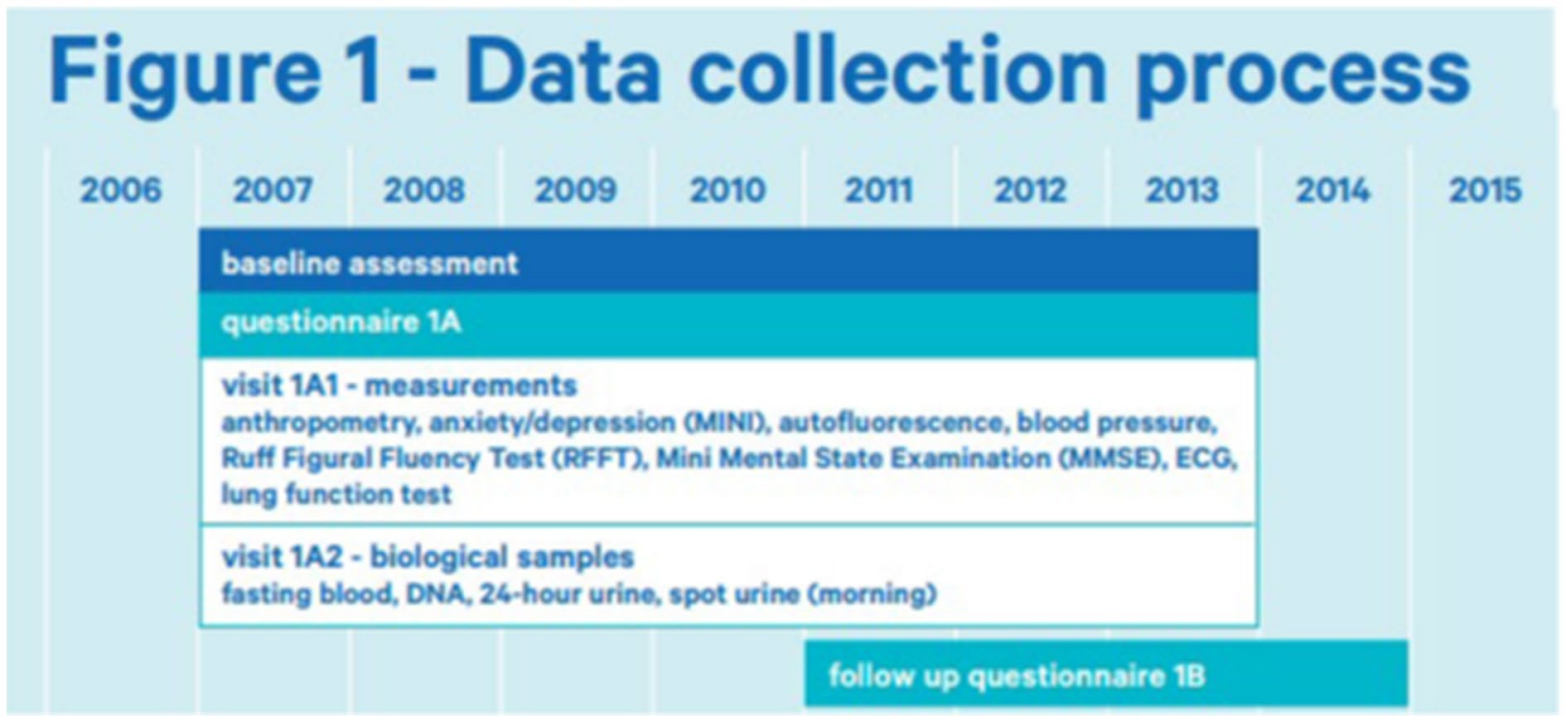
Data collection process of lifelines (14).

This study was performed using the Dutch Lifelines Cohort Study (13). Lifelines is a multi-disciplinary prospective population-based cohort study examining in a unique three-generation design the health and health-related behaviors of 167,729 persons living in the north of the Netherlands. It employs a broad range of investigative procedures in assessing the biomedical, socio-demographic, behavioral, physical and psychological factors that contribute to the health and disease of the general population, with a special focus on multi-morbidity and complex genetics. The first participants were included in 2006 and will be followed for at least 30 years. The baseline assessment took place from 2007 to 2013 and included questionnaires (1A) as well as different measurements (1A1) and biological samples (1A2) (Figure 1). As part of the assessment, participants were asked to fill out surveys, with follow-up surveys approximately once every 1.5 years. The first follow-up questionnaire (1B) was sent from 2011 to 2014. For more information on the Lifelines Cohort please see the study by Scholtens et al. (13). The Lifelines initiative has been made possible by a subsidy from the Dutch Ministry of Health, Welfare, and Sport, the Dutch Ministry of Economic Affairs, the University Medical Center Groningen (UMCG), Groningen University, and the provinces in the north of the Netherlands (Drenthe, Friesland, and Groningen).

The Lifelines Cohort Study is performed in accordance with the principles of the Declaration of Helsinki and in accordance with the University Medical Center Groningen (UMCG) research code. All participants provided written informed consent. The Lifelines protocol was approved by the UMCG Medical ethical committee under number 2007/152.

### Variables

In this study, we included data from questionnaires at moment 1A and 1B, and data from measurements at visit 1A1. For several variables, items out of the questionnaire from both moments were collected. For example, the presence of cancer was asked at both moments 1A and 1B. For these variables, a new variable was created that combined the information from both points in time. Please see the Lifelines website for the exact formulation of each variable, the answer options, or the calculation methods (15).

### Model outcome

Tinnitus presence (1B) was assessed with the following question: ‘*Do you hear ringing or whistling in your ear/ears?’,* with the answer options “*No never, “Yes, sometimes,”* or “*Yes, always*.” In this study, participants were defined as having tinnitus when they answered: “*Yes, always.”* If participants answered “*No, never”* or “*Yes, sometimes”* they were defined as not having tinnitus.

### Candidate predictors

We included 24 different variables over different domains. These variables were considered as candidate predictors of tinnitus experience based on the literature and expert opinion (discussion by MR, AL, and IS) (8, 9).

#### Demographic

The following demographic variables were assessed: educational attainment (low, middle, or high) (1a),age (if available at 1B or else at 1A), and sex (male or female).

#### Mental health

##### Subjective mental health

The following subjective variables were assessed within the mental health domain: presence or history of anxiety disorders (1A & 1B), depression (1A & 1B), and burnout (1A & 1B). The variable anxiety disorder was a combination of the presence or history of either/or anxiety disorder (1A), social phobia (1A), agoraphobia (1A & 1B), and panic disorder (1A & 1B).

##### Symptom checklist

We used the Symptom Checklist (SCL-90) to assess somatic complaints at 1B (16, 17). The list consists of 12 questions, with answer options on a 5-point Likert scale (1 not at all to 5 very much). The sum score was calculated. Total sum scores ranged between 12 and 60, in which higher scores indicated a higher severity of somatic symptoms.

##### Emotional affect

The positive and negative affect schedule (PANAS) was used to assess emotional affect (18) (1A). The PANAS consists of 20 items, 10 for a positive affect, 10 for a negative affect. All questions had to be answered with 5-point Likert scales (1 not at all to 5 extremely). Scores ranged from 10 to 50, with lower scores indicating lower levels of a positive or negative affect and higher scores indicating higher levels of a positive or negative affect.

#### Personality

Personality was assessed with the NEO personality inventory (NEO-PI-R) (1A) (19, 20). This self-report tool measures the five most significant aspects of personality: neuroticism, extraversion, openness, agreeableness, and conscientiousness with 240 items on a 5-point Likert scale. At baseline, Lifelines used two shorter versions of the NEO-PI-R, which focused on conscientiousness, extraversion, and neuroticism. These domains were also assessed in this model.

#### Quality of sleep

The Pittsburgh sleep quality index (PSQI) was used to measure sleep quality at 1B (21). The questionnaire measures sleep quality and disturbances over a 1-month time interval. In 19 questions, seven component scores are assessed. The total score is a sum score of the component scores. This was dichotomized by Lifelines into either a good (PSQI >5) or a bad quality of sleep (PSQI ≤5) (22).

#### Cognition

Cognition was measured by the Ruff Figural Fluency Test (RFFT) at 1A (23). The RFFT measures the cognitive function domain of non-verbal fluency. The test is made up of five parts. In these parts the patient is presented with a different pattern of dots. A fixed time period is set, and the patient is asked to draw as many unique designs as possible. The number of unique designs is a measure of non-verbal fluency and was used as a predictor.

#### Hearing health

The following variables were assessed within the ear domain: disturbance of daily life because of hearing loss (1B) and the use of hearing aids (1B).

#### Cardiovascular disease

We combined several variables to create two predictor candidates. The first was cardiovascular disease, which included the presence or positive medical history of either/or hypertension (1A), high cholesterol (1A), and diabetes (1A & 1B). The second was major adverse cardiac events (MACE), which included the presence or positive medical history of either/or heart attack (1A & 1B), stroke (1A & 1B), carotid stenosis (1A), stenosis for which an angioplasty or bypass surgery was necessary (1A), angioplasty (1A & 1B), atherosclerosis (1A), and claudication (1B).

#### Cancer

The presence or history of cancer was scored as one variable based on a positive answer to this question at either baseline or follow-up (1A and 1B).

#### Neurological disorders

The presence or history of neurological disorders was based on the presence or medical history of Parkinson’s (1A) and/or multiple sclerosis (1A).

#### Physical activity

The Short Questionnaire to Assess Health-enhancing physical activity (SQUASH) was used to assess physical activity. The sum score was used to categorize participants into meeting the recommended Dutch level of exercise as determined by the Dutch Health Board (24, 25) (1A).

#### General health

The question: “*How would you rate your health, generally speaking” (excellent, very good, good, fair, or poor)* was used to assess general health at moment 1B. This question is part of the RAND-36 Quality of Life questionnaire (26).

#### Physical status

The following measurements of physical status were performed at baseline and included as candidate predictors: BMI and mean arterial pressure (1A1).

### Statistical analyzes

Data cleaning was conducted in SPSS version 27 (27). Other statistical analyzes were performed in R studio (version 22.02.0) using the glmnet and caret packages (28, 29). A sample size calculation was performed in R with the pmsampsize package (30).

Frequencies and percentages of categorical variables were calculated. For continuous data, normality was assessed. Normally distributed data was presented as means with standard deviation (SD). Non-normally distributed data were presented as medians with interquartile range (IQR).

Missing data (Missing at random) was imputed with multiple imputation, with 30 imputation sets. All missing data were imputed, except for the missing data of the original tinnitus question, of which 22,829 cases were missing. These were excluded from the data; therefore, the analyzes were formed only on those data of which an answer to the original tinnitus question was known.

A multivariable elastic net logistic regression model was used to develop the prediction model. Elastic net is a combination of Lasso selection and ridge penalization (31). A 10-fold cross-validation was used to minimize cross validating deviance, by determining the optimal tuning parameters (alpha and lambda values) of the model.

An elastic net model was fitted on each of the 30 imputations set. Estimates of the optimal tuning parameters (alpha and lambda) and model performance measures were calculated for each model. The mean was calculated for each of those in the final model, which is presented in this manuscript by Rubin’s Rules. Estimates were included in the final model if the value was >0.001 in positive numbers or > −0.001 in negative numbers 16 or more times. The model was internally validated by 10-fold cross-validation.

The performance of the model and the internal validation were assessed with the RMS package (32). Pseudo R2 and Brier score were calculated as overall performance measures. The c-statistic was calculated to assess discrimination and the calibration intercept and slope were calculated as calibration measures.

## Results

### Baseline characteristics

Data was collected from 151.113 participants. Of those, 83.756 (55.4%, 22.829 missing) answered “*no, never”* to the question “*Do you hear ringing or whistling in your ear/ears*,” whereas 31.163 (20.6%, 22,829 missing) answered “*yes sometimes*,” and 7,965 (5.3%, 228,229 missing) answered “*yes always*.” A total of 112.884 participants did not answer the question about tinnitus; therefore, the total number included in the analyzes was 122.884. Of those, according to our definition, 7,965 (6.5%, 0 missing) experienced tinnitus and 114.919 did not (93.5%, 0 missing) (Table 1).

**Table 1:** Baseline characteristics on tinnitus.

Variable	*N*	%
*Do you hear ringing or whistling in your ear/ears?*		
No never	83,756	55.4
Yes sometimes	31,163	20.6
Yes always	7,965	5.3
Missing	228,229	18.7
*Tinnitus* ^a^		
No	114,919	93.5
Yes	7,965	6.5

a Tinnitus as to our definition.

The missing data were not included in the analyzes.

The majority of the participants were women (72.862, 59.3%, 0 missing), whereas, of those with tinnitus, the majority were men 4,557 (57.2%, 0 missing). The mean age was 45.0 years (SD 12.8), the mean age of participants without tinnitus was 44.5 years of age (SD 12.7) and of the participants with tinnitus, the mean age was 52.6 years (SD 11.6). Most participants were not disturbed in their daily life because of hearing loss (106.285, 86.5%, 384 missing). However, 663 (0.5%, 384 missing) participants were severely limited in their daily life because of hearing loss, and 15.552 (12.7%) were a bit limited. See Table 2 for the baseline characteristics of the analyzed data.

**Table 2:** Baseline characteristics.

Variable	Total		Tinnitus No		Tinnitus Yes	
	N	%	N	%	N	%
*Sex*						
Male	50,022	40.7	45,465	39.6	4,557	57.2
Female	72,862	59.3	69,454	60.4	3,408	42.8
Missing	0	0	0	0	0	0
*Educational attainment*						
Low	35,682	29.04	32.584	28.4	3,098	38.9
Middle	48,164	39.2	45.496	39.6	2,668	33.5
High	37,675	31.1	35.599	31.0	2076	26.1
Missing	1,363	1.11	1,240	1.1	123	1.5
*Hearing aid (do you need a hearing aid?)*						
Yes	4,052	3.3	2,876	2.5	1,176	14.8
No	118,518	96.5	11,769	97.3	6,749	84.7
Missing	314	0.3	40	0.5	315	0.3
*Disturbance of daily life because of hearing loss*						
Yes, severely limited	663	0.5	362	0.3	301	3.8
Yes, a bit limited	15,552	12.7	12,037	10.5	3,515	44.1
No, not limited at all	106,285	86.5	102.168	88.9	4,117	51.7
Missing	384	0.3	352	0.3	32	0.4
*Squash exercised norm*						
Yes	63,239	51.5	588,559	51.0	4,680	58.8
No	49,544	40.3	46,900	40.8	2,644	33.2
Missing	10,101	8.2	9,460	8.2	641	8
*PSQI quality score*						
Good sleep quality	87,619	71.3	82.450	71.7	5,169	64.9
Poor sleep quality	31,399	25.6	28.920	25.2	2,479	31.1
Missing	3,866	3.2	3,549	3.1	317	4.0
*Rand general health score*						
Excellent	9,339	7.6	8,951	7.8	388	4.9
Very good	30,882	25.1	29.423	25.6	1,459	18.3
Good	70,804	57.6	66.012	57.4	4,792	60.2
Fair	10,756	8.8	9,571	8.3	1,185	14.9
Poor	755	0.6	641	0.6	114	1.4
Missing	348	0.3	321	0.3	27	0.3
*Burnout*						
Yes	12,495	10.2	11.417	9.9	1,078	13.5
No	110,389	89.8	103.502	90.1	6,887	86.5
Missing	0	0	0	0	0	0
*Depression*						
Yes	12,845	10.5	11,749	10.2	1,096	13.8
No	110,039	89.6	103.170	89.8	6,869	86.2
Missing	0	0	0	0	0	0
*Cancer*						
Yes	5,800	4.7	5,197	4.52	603	7.57
No	116,978	95.2	109,623	65.39	7,355	92.34
Missing	106	0.09	99	0.09	7	0.09
*Anxiety*						
No	115,685	94.1	108.309	94.2	7,376	92.6
Yes	7,199	5.9	6,610	5.8	589	7.4
Missing	0	0	0	0	0	0
*Neurologic disease*						
No	122,454	99.7	114,521	99.7	7,933	99.6
Yes	430	0.4	398	0.3	32	0.4
Missing	0	0	0	0	0	0
*Risk factor Cardiovascular disease*						
No	85,849	69.86	81.238	70.7	4,611	57.9
Yes	37,033	30.1	33,680	29.3	3,353	42.1
Missing	2	0.002	1	0.0009	1	0.01
*Major adverse cardiovascular event*						
No	118,822	96.7	111.351	69.9	7,471	93.8
Yes	4,062	3.3	3,568	3.1	494	6.2
Missing	0	0	0	0	0	0
*BMI*						
Median (IQR)	25.4	5.09	25.34	5.06	26.0	4.8
Missing	77	0.06	73	0.06	4	0.05
*BP average arterial mean*						
Mean (SD)	93.16	10.24	92.98	10.19	95.8	10.6
Missing	113	0.09	101	0.09	12	0.15
*Age*						
Mean (SD)	45.01	12.82	44.5	12.74	52.6	11.58
Missing	0	0	0	0	0	0
*Panas positive*						
Median (IQR)	36	5	36	5	35	6
Missing	3,443	2.8	3,215	2.8	228	2.9
*Panas negative*						
Median (IQR)	20	7	20	7	20	7
Missing	3,443	2.2	2,313	2.01	169	2.12
*RFFT som unique design*						
Median (IQR)	82	33	82	32	76	33
Missing	45,254	36.8	42,129	36.7	3,125	39.2
*SCL somatic*						
Median (IQR)	15	4	15	4	15	6
Missing	7,553	6.1	6,805	5.9	748	9.4
*Neuroticism*						
Median (IQR)	26	9	26	9	26	10
Missing	10.493	8.5	9,158	8.0	1,335	16.8
*Extraversion*						
Median (IQR)	36	12	37	12	36	15
Missing	10,573	8.6	9,236	8.04	1,337	16.8
*Conscientiousness*						
Median (IQR)	46	7	46	7	46	9
Missing	10.495	8.5	9,160	8.0	1,335	16.8

### Model

The mean alpha of the elastic net models was 0.197 and the lambda was 0.046. Nine variables with 10 categorical sub-variables made up the final model, all other variables were removed from the model after shrinkage (Table 3). The following variables were selected in the final model: male sex (ref = female, OR 1.2982), no hearing aids (ref = yes, OR = 0.6811), hearing limitations (a bit, ref. = severely limited, OR = 1.4903), hearing limitations not at all (ref = severely limited, OR = 0.3879), mean arterial blood pressure (OR = 1.0013), a bad quality of sleep on the PSQI (Ref = good quality of sleep, OR = 1.00571), fair score on the Rand general health (ref = excellent score, OR = 1.07358), SCL somatic sum score (OR = 1.0736), CVD risk factors (ref = no CVD risk factors, OR = 1.0027), and age (OR = 1.01714).

**Table 3:** Coefficients of the apparent performance model.

Variable	Coefficient	Odds ratio
Intercept	−3.029	0.0484
Sex, female	Reference	
Sex, male	0.261	1.2982
Hearing aid, yes	Reference	
Hearing aid, no	−0.384	0.6811
Hearing limitation, severely limited	Reference	
Hearing limitation, a bit	0.399	1.4903
Hearing limitation, not at all	−0.947	0.3879
Mean arterial blood pressure	0.0013	1.0013
PSQI Good quality of sleep	Reference	
PSQI Bad quality of sleep	0.0057	1.0057
Rand general health, excellent	Reference	
Rand general health, fair	0.071	1.0736
SCL somatic sum score	0.0189	1.0191
CVD risk factors, no	Reference	
CVD risk factors, yes	0.0027	1.0027
Age	0.017	1.0017

### Discrimination

Discrimination expresses how well the risk model distinguishes between cases and non-cases. The area under the curve (AUC) of the model was 0.789 in the apparent performance.

### Calibration

**Figure 2:**
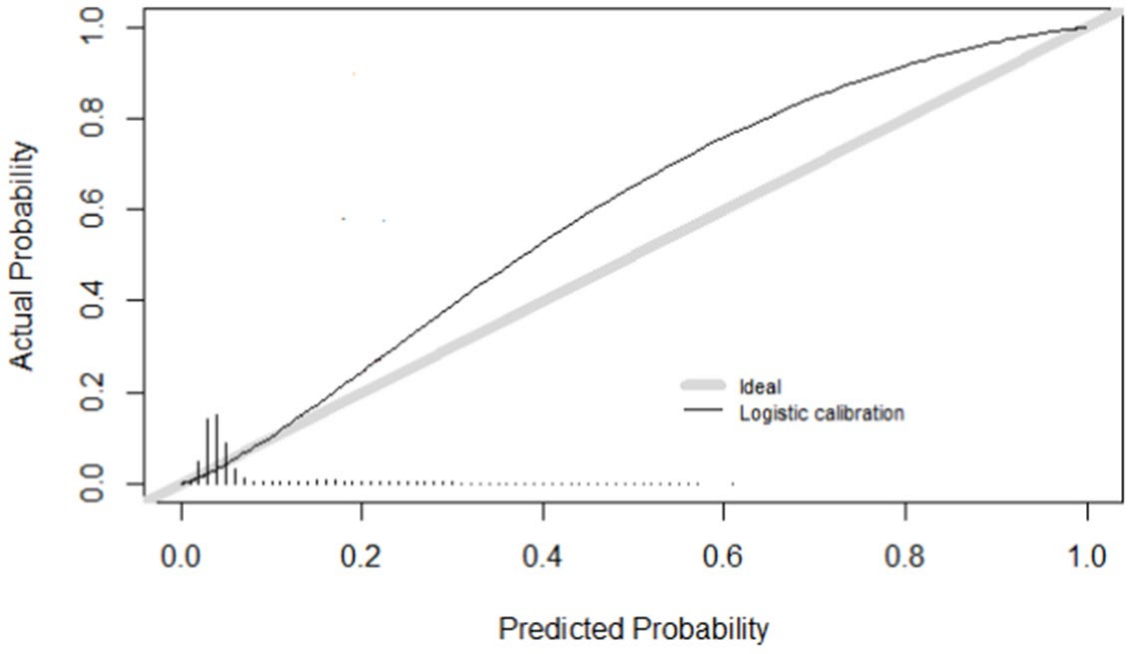
Calibration curve.

Calibration refers to the level of agreement between calculated risks and observed outcomes. Figure 2 shows the calibration curve of the model. Calibration was expressed as an intercept of 0.75, with a slope of 1.315 (Table 4). The R2 was 0.155 and the Brier score was 0.056.

**Table 4:** Model performance measures.

Model performance measure	Apparent performance model	Internally validated model
*Overall performance*		
Pseudo R2	0.155	0.158
Brier	0.056	0.056
*Discrimination*		
C-statistic	0.789	0.787
*Calibration*		
Intercept	0.750	0.634
Slope	1.315	1.268

### Internal validation

We internally validated the model with 10-fold cross-validation. Figure 2 shows the calibration curve. Of the internally validated model, the R2 was 0.158 and the Brier score was 0.056.

## Discussion

We developed and internally validated a model on the experience of tinnitus. We created this model in a large representative dataset of the adult Dutch general population (122.884 participants were included in our model) (13). We developed a prediction model and internally validated it to assess the performance. The final model included nine different predictors, out of 24 candidate predictors.

One of the challenges in making a tinnitus prediction model, and in tinnitus research, are the multiple different definitions of tinnitus (3, 33, 34). Amongst others, one can differentiate between having tinnitus based on cutoffs for frequency and duration of the experienced sound, but also on the experienced impact. Differences in used cutoffs and definitions result in differences in the outcomes of studies concerning tinnitus. This is also the most important limitation of our study. The exact wording of the question asking about the experience of tinnitus was” *Do you hear ringing or whistling in your ear/ears*?” The answer options were: “*No, never*,” “*Yes, sometimes*,” or “*Yes, always*,” and categorized in tinnitus experience yes or no. Besides this, tinnitus is not limited to merely a ringing or whistling sound as indicated in the question. Those examples might have confused participants or resulted in a selection of those answering positive to the question and not including people having other kinds of tinnitus sounds (35).

Another limitation of our study is the use of variables based on multi-item questionnaires. As explained in a recent research paper by van Royen et al., including costly or time-intensive variables in prediction models is one of the reasons why the adaption of prediction models fails in clinical practice (36). In the current model, we used different time-intensive and not readily available assessments of personality, emotional affect, verbal fluency, somatic complaints, and sleep quality. However, most of these variables were shrunken out of the final model, in which only the SCL somatic sum score and the PSQI were included. We were aware of this limitation before we started the development of this model, but decided to include these variables since we wanted to approach the concept of these predictors. Future research should consider several, more accessible derivates of these variables to maximize clinical applicability. This model is of added value for research purposes as well as (preventative) policies. Finally, model performance of the internal validation might be slightly optimistic due to using nested cross-validation rather than bootstrapping (37).

In a recent systematic review of prediction models, we noticed that demographic factors were mostly used as predictors in the final models on tinnitus experience. Whereas comorbidities were mostly used as predictors in models on tinnitus impact (11). In the current model on tinnitus experience, we found both demographic factors and comorbidities to be predictors. Of the nine predictors in the final model, two are hearing-related comorbidities. Although there is debate in the literature on this issue, it should be emphasized that hearing-related difficulties are widely seen as causal to experiencing tinnitus (9, 38). The outcome of the present study is in line with this statement.

Future research that focuses on the creation of a prediction model on tinnitus impact would be helpful for clinical practice. In this study, we did not perform an external validation of our prediction model. This should be considered for future studies to assess the model’s accuracy, reproducibility, and generalizability in a different dataset (10, 39).

## Conclusion

In this study, we developed and internally validated a prediction model on tinnitus experience. The predictors included were the male sex (compared to the female sex), the use of hearing aids (compared to no use), the presence of hearing limitations, mean arterial blood pressure, a bad quality of sleep (compared to a good quality sleep), a fair subjective opinion of their general health (compared to an excellent opinion of general health), somatic complaints, the presence or history of cardiovascular risk factors (compared to no presence of history), and age. This manuscript stresses the potential incremental value of comorbidities, especially hearing-related comorbidities for the purpose of predicting tinnitus.

## Data availability statement

Data may be obtained from a third party and are not publicly available. Researchers can apply to use the Lifelines data used in this study. More information about how to request Lifelines data and the conditions of use can be found on their website: https://www.lifelines.nl/researcher/how-to-apply.

## Ethics statement

The studies involving human participants were reviewed and approved by UMCG Medical ethical committee under number 2007/152. The patients/participants provided their written informed consent to participate in this study.

## Author contributions

MR, AS, RS, and IS participated in the conception or design of the work. Data analysis and interpretation was performed by MR, AS, MS, and IS. MR drafted the article. AS, RS, MS, and IS critically revised the article. All authors contributed to the article and approved the submitted version.

## Funding

The research was funded by Cochlear, the funder did not have any role in the study design, collection, analyzes, or interpretation of the results. The Lifelines initiative has been made possible by a subsidy from the Dutch Ministry of Health, Welfare, and Sport, the Dutch Ministry of Economic Affairs, the University Medical Center Groningen (UMCG), Groningen University, and the provinces in the north of the Netherlands (Drenthe, Friesland, and Groningen).

## Conflict of interest

The authors declare that the research was conducted in the absence of any commercial or financial relationships that could be construed as a potential conflict of interest.

## Publisher’s note

All claims expressed in this article are solely those of the authors and do not necessarily represent those of their affiliated organizations, or those of the publisher, the editors and the reviewers. Any product that may be evaluated in this article, or claim that may be made by its manufacturer, is not guaranteed or endorsed by the publisher.
